# Diagnostic Management of Pregnant Women With Suspected Pulmonary Embolism

**DOI:** 10.3389/fcvm.2022.851985

**Published:** 2022-03-16

**Authors:** Helia Robert-Ebadi, Grégoire Le Gal, Marc Righini

**Affiliations:** ^1^Division of Angiology and Hemostasis, Geneva University Hospitals and Faculty of Medicine, Geneva, Switzerland; ^2^Department of Medicine, Ottawa Hospital Research Institute, University of Ottawa, Ottawa, ON, Canada; ^3^EA3878 University of Brest, Brest, France

**Keywords:** pulmonary embolism, diagnostic strategy, D-dimer, clinical probability, pregnancy, computed tomography pulmonary angiography, ventilation-perfusion lung scan

## Abstract

Pulmonary embolism (PE) is one of the most common causes of severe morbidity and mortality during pregnancy. PE diagnosis during pregnancy remains a true challenge for all physicians, as many of the symptoms and signs associated with PE are often reported during physiological pregnancy. The fear of missing a PE during pregnancy leads a low threshold of suspicion, hence to a low prevalence of confirmed PE among pregnant women with suspected PE. This means that most pregnant women with suspected PE do not have the disease. Until recently, international guidelines suggested thoracic imaging in all pregnant women with suspected PE. Two recent prospective management outcome studies based on clinical probability assessment, D-dimer measurement, venous compression ultrasonography of the lower limbs (CUS) and computed tomography pulmonary angiography (CTPA) proved the safety of such strategies, with a very low failure rate. For the first time, these studies also demonstrated that the association of a clinical prediction rule and D-dimer measurement allowed a safe exclusion of PE in a significant proportion of pregnant women, without the need for radiating imaging tests. These two prospective studies pave the way to further improvements in the diagnostic strategies. Indeed, both specific clinical prediction rules and possibly D-dimer cutoffs adapted to pregnant women could help to further reduce the proportion of patients needing thoracic imaging. As an imaging test will still ultimately be necessary in a significant proportion of women, further technical advances in CT scans protocols could reduce the radiation dose to both the fetus and the mother, an important step to reassure clinicians. Finally, educational efforts should be encouraged in the future to pass the challenge of implementing these validated diagnostic strategies in everyday clinical practice.

## Introduction

Pregnancy represents a period at risk of venous thromboembolism (VTE) in women of child bearing age who are otherwise at low risk of developing VTE. The overall incidence of VTE is estimated at 1/1'000 pregnancies, and in western countries, pulmonary embolism (PE) remains a leading cause of maternal mortality (1, 2). The risk is highest during the third trimester and the 6 to 12 weeks following delivery (3).

### Clinical Suspicion of PE During Pregnancy

PE diagnosis during pregnancy remains a true challenge for physicians, as many of the symptoms and signs frequently reported during physiological pregnancy–such as shortness of breath or tachypnea–may also suggest the diagnosis of PE (4). This is also true for symptoms and signs suggestive of the presence of a deep vein thrombosis (DVT). Indeed, lower limb pain and/or edema is often reported by pregnant women, especially during the second half of pregnancy (5). It is therefore particularly difficult to set a threshold between what can be considered physiological and what should raise the suspicion of VTE and lead to further investigations.

### Prevalence of Confirmed PE Among Pregnant Women With Suspected PE

Although VTE risk is increased 7 to 10-fold during pregnancy compared to age-matched controls, the absolute incidence remains low (around 1/1'000) (6). Nevertheless, the fear of missing a PE during pregnancy leads to a low threshold to suspect the disease. This results in a very low prevalence of confirmed events among suspected patients even in the setting of clinical trials. Compared to the PE prevalence observed in diagnostic trials outside pregnancy (10–20% depending on geographic location) (7, 8), the reported PE prevalence in pregnant women is much lower at around 2–7% (4, 9, 10). In other words, the vast majority of pregnant women in whom PE is suspected do not have PE. Therefore, the main focus of diagnostic strategies is–even more than outside pregnancy–to *rule out* the diagnosis. This is an important information to bear in mind when considering the use of radiating imaging tests, and highlights the necessity of finding alternative strategies to minimize the proportion of pregnant women who need chest imaging.

## Diagnostic Approach to Pregnant Women With Suspected Pulmonary Embolism

Historically, all pregnant women with suspected PE underwent a thoracic imaging test. In the 1990's, ventilation-perfusion scintigraphy (V/Q scan) was assessed in the PIOPED trial in the general population of patients with suspected PE (11), and perfusion only scans were rapidly adopted in clinical practice in pregnant women, albeit without previous scientific validation. Interestingly, in a study assessing the appropriateness of diagnostic management of patients with suspected PE, pregnancy was by far the strongest predictor of inappropriate management: 69% of pregnant women with suspected PE were indeed not appropriately managed (12). The lack of solid prospective data specific to this patient population is highly likely to have contributed to this observation published in 2006 (12).

Since then, two prospective management outcome studies have assessed two different strategies, and were published in 2018 (9) and 2019 (10). These two studies represent the first prospective scientific validation of PE diagnostic strategies during pregnancy and will be described in detail below.

### Is There Any Clinical Pre-test Probability Assessment Tool I Could Use During Pregnancy?

In patients with suspected PE, the assessment of clinical pre-test probability (PTP) is the first step of all current diagnostic management strategies and is strongly encouraged in international guidelines (13, 14). It allows identifying a subgroup of patients with a low prevalence of the disease in whom a negative D-dimer safely rules out PE without imaging. It is also sometimes used for the final diagnostic interpretation of V/Q scan results.

**Figure 1:**
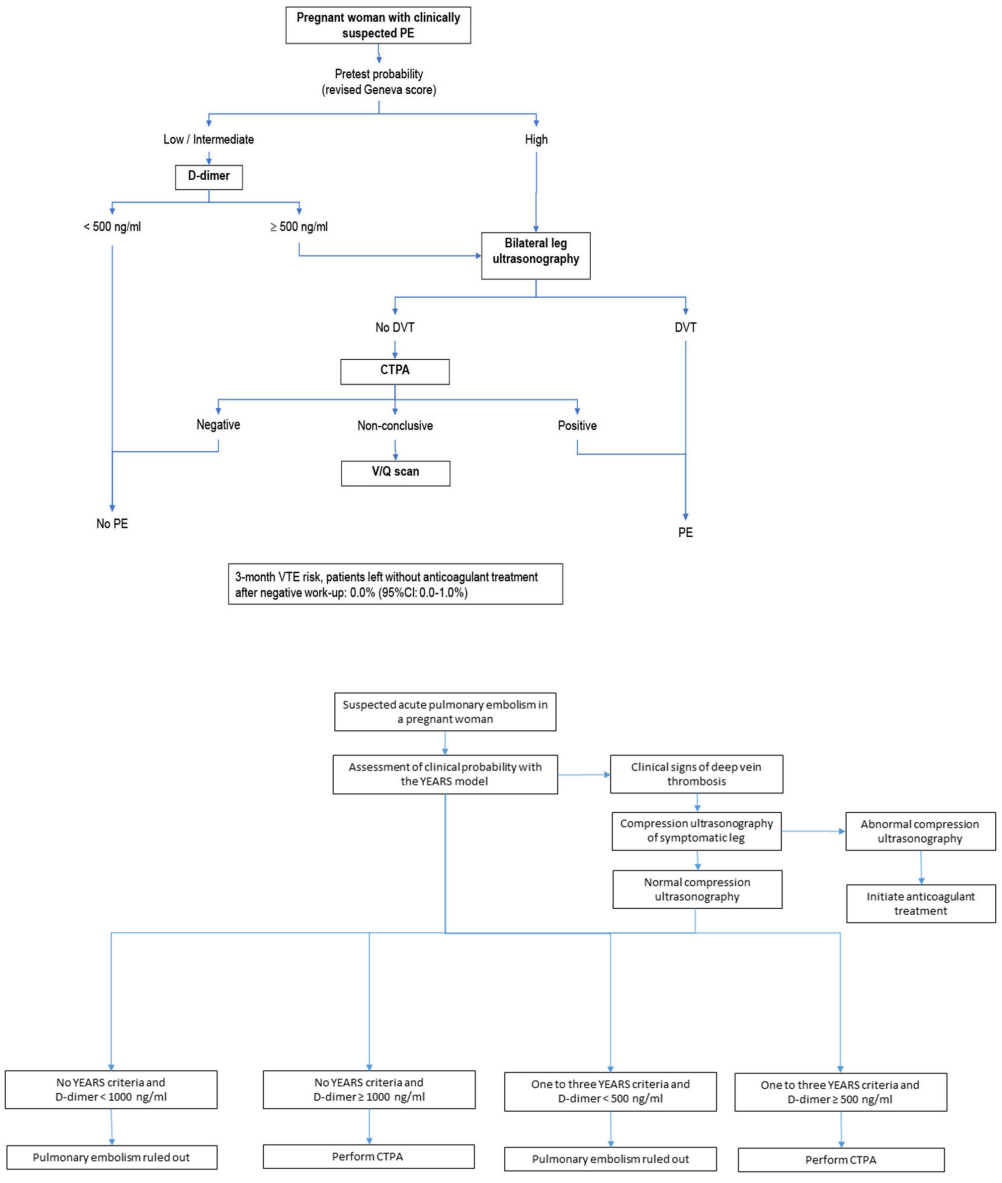
The CT-PE pregnancy and the pregnancy-adapted YEARS diagnostic algorithms (9, 10).

Available clinical decision rules (CDRs) have not been derived or validated in pregnant women (15). This has been one of the reasons brought by some physicians for not using D-dimer in this setting. The CT-PE pregnancy and ARTEMIS studies assessed diagnostic strategies in the specific setting of pregnant women with suspected PE (9, 10). These studies used two different PTP assessment tools–the Geneva score and a pregnancy-adapted YEARS model- that had not been previously derived nor validated in a pregnant population. Nevertheless, these two CDRs both proved efficient and accurate in integrated diagnostic algorithms (see Figure 1) (9, 10).

Further steps were taken in the validation process of pregnancy-adapted CDRs with the external validation of the YEARS model in the CT-PE pregnancy population, confirming the safety of this model in a second cohort of patients (16). Moreover, a novel PTP assessment tool-the Pregnancy-Adapted Geneva Score (PAG score)–was recently developed. The PAG score contains only *objective* items that are all relevant to pregnant women (see Table 1) (17). It allows classifying pregnant women with suspected PE in three categories of PTP that correspond to increasing prevalence of the disease (see Table 1). However, before advocating its large scale use in clinical practice, the PAG score needs to be prospectively validated.

**Table 1:** The Pregnancy-Adapted Geneva score for assessment of pre-test clinical probability of PE in pregnant women (17).

**The Pregnancy-Adapted Geneva score**	
**Item**	**Points**
Age 40 years and older	+1
Surgery (under GA) or lower limb fracture in past month	+2
Previous DVT or PE	+3
Unilateral lower limb pain	+3
Haemoptysis	+2
Pain on lower limb palpation and unilateral oedema	+4
Heart rate > 110 bpm	+5
Maximal point number	20
**Points**	**PTP category**	**PE prevalence in development cohort**	**95% CI**
0–1	Low	2.3%	1.0–4.9 %
2–6	Intermediate	11.6%	6.9–18.9%
≥7	High	61.5%	35.5–82.2%

### Should I Use D-Dimer to Exclude PE During Pregnancy?

In clinical practice, D-dimer testing tends to be more often skipped in pregnant women than in the general population because of the knowledge of gradually increasing D-dimer levels during pregnancy. Physicians therefore tend to consider D-dimer as “useless” in this setting. Another reason which likely contributes to a reluctance to use D-dimer during pregnancy is, as said above, the lack of clinical PTP assessment scores specifically developed and validated in pregnant women. Finally, the safety of excluding PE by a negative D-dimer during pregnancy has been challenged by some authors (18).

Nevertheless, the safety of a negative D-dimer associated with a non-high/unlikely PTP to exclude PE without imaging is widely accepted outside pregnancy (7, 8, 19). There is no biological rationale which could support the hypothesis of a lower sensitivity of D-dimer during pregnancy. The CT-PE pregnancy and ARTEMIS studies have both confirmed the safety of excluding PE during pregnancy by a negative D-dimer test in stepwise diagnostic algorithms (see Figure 1). The number of patients remains limited in these studies and needs to be extended and enriched by data from future prospective trials. Nonetheless, a recent meta-analysis reported a high negative predictive value of D-dimer to exclude PE during pregnancy. The pooled estimate values were indeed 99.5% for sensitivity (95% CI 95.0–100.0%) and 100% for negative predictive value (95% CI 99.19–100.0%) (20).

While awaiting additional data, the clinician should bear in mind that the percentage of pregnant women in whom PE diagnosis can be safely excluded by a non-high PTP and negative D-dimer without any additional tests (CUS or thoracic imaging) was of 12% in the CT-PE-pregnancy study, which corresponds to a number of patients needed to test to exclude one PE of 8.3 (9). In a setting where minimizing radiating tests is a central concern, this efficiency is not negligible. Avoiding radiation exposure by a simple blood test in 1 out of 8 pregnant women with suspected PE is already highly appealing. Of note, although the risk of developing PE is highest during the post-partum and the third trimester, the chances of obtaining a negative D-dimer test decreases with advancing pregnancy. In the CT-PE pregnancy study, the proportion of negative D-dimer results was 25% during the first trimester, 11% during the second trimester, and 4% during the third trimester.

The ARTEMIS study also showed a high efficiency of the diagnostic algorithm to avoid thoracic imaging (39%). However, the non-invasive strategy tested in this study was not solely based on PTP and D-dimer but included the pre-exclusion of DVT by lower limb CUS in patients with lower limb symptoms, and so represented a more complex selection of “low-risk” women in whom a higher D-dimer cutoff was used to exclude PE (see Figure 1) (10). When pooling all the available evidence to address this question, the recent meta-analysis mentioned above demonstrated an overall efficiency of 34% of D-dimer to safely exclude PE (95% CI 15.9–55.23%) (20). Giving the chance to a pregnant woman with suspected PE to avoid a radiating test should therefore not be neglected, and in spite of the controversies in international guidelines (21), we believe that the use of D-dimer in this setting should be strongly encouraged.

### Should I Refer My Patient for Lower Limb Compression Ultrasound Before Chest Imaging?

The information required to answer this question is provided by the CT-PE pregnancy and the ARTEMIS studies. Bilateral lower limb compression ultrasound (CUS) was indeed part of the diagnostic strategies of both studies (see Figure 1). In the CT-PE pregnancy study, CUS was performed in 75% of the overall population, and proximal DVT was diagnosed in 2% of patients without leg symptoms and 9% of patients with leg symptoms (9). In the ARTEMIS study, CUS was performed in 88% of the overall population, and DVT was confirmed in 1% of patients without leg symptoms and 7% of patients with leg symptoms (10). CUS seems thus mainly useful in pregnant women with lower limb symptoms (lower limb pain and or edema). Nevertheless, focusing again on the need to maximize the number of avoidable radiating tests rather than on cost-effectiveness, the yield of 1–2% avoided radiating tests provided by screening pregnant women with CUS is considered worthwhile by some. Depending on the structure of medical care facilities, obtaining a CUS can however be more challenging that obtaining a CTPA and represents an obstacle to the implementation of a sequential testing strategy (15). Another potential limitation is the difficulty to assess iliac veins and diagnose isolated iliac DVT, which is however mainly a problem in pregnant women with suspected DVT with no concomitant PE suspicion. Interestingly, international guidelines currently recommend bilateral CUS in pregnant women with suspected PE in whom PE diagnosis could not be excluded by the combination of PTP and D-dimer, in whom further testing is needed, before pursuing to chest imaging (13).

### What Chest Imaging Modality Should I Choose in Pregnant Women and Why?

In spite of all the efforts described above to minimize chest imaging, a significant proportion of pregnant women with suspected PE (around 2/3) will ultimately need thoracic imaging in their diagnostic management (20). Radiation exposure to the mother and to the fetus are both matters of concern (22). CTPA and V/Q scan are the two chest imaging modalities studied on a large scale basis outside pregnancy. Due to its high diagnostic accuracy and accessibility, CTPA has become the new “gold standard” for the diagnosis of PE and is the most widely used test in clinical practice (23). Another advantage of CTPA is the possibility to identify an alternative diagnosis such as aortic dissection, pneumonia, pneumothorax, which can be missed by V/Q scan.

This has even led to major concerns about over-testing and over-diagnosis, which are beyond the scope of the present paper. In pregnant women, radiation exposure and the rate of non-diagnostic tests remain the two central matters of concern.

A recent meta-analysis comparing all the available data on these two imaging techniques in pregnant women could not conclude on the relative risk of radiation of CTPA vs. V/Q scan due to lack of homogeneity in the calculation methods and the scan protocols used (22). The important message conveyed by this work is however that all reported radiation measurements for both tests were clearly below the commonly accepted harmful threshold of 100 mGy, in spite of the inclusion of older studies preceding the implementation of adapted imaging protocols in pregnant women (22). Moreover, as previously stated by scientific societies and experts, the risks associated with either test is by far lower than the potential risks of inappropriate diagnostic management leading to a missed diagnosis (with the risk of death) or to an unjustified anticoagulant treatment based on a “clinical” diagnosis (with the risk of bleeding and the long term consequences on delivery and subsequent pregnancies of a unduly confirmed PE) (24, 25). Regarding the proportion of inconclusive tests, the pooled rates of non-diagnostic results were similar between CTPA (14%) and V/Q scan (12%), but the range of reported rates was very broad (0–57% for CTPA and 1–40% for V/Q scan) across the included studies (22).

The technical evolution of CTPA has considerably decreased radiation exposure, and pregnancy-adapted CTPA protocols include a reduced anatomical coverage of the scan and reduced kilovoltage. These specific protocols also include a high-concentration, high-volume and high-rate of injection of contrast media followed by saline flush as well as shallow inspiration breath-hold to avoid a Valsalva manoeuver, in order to optimize arterial opacification and avoid non-diagnostic tests (26). The technical evolution of nuclear medicine imaging modalities, including tomographic lung scintigraphy (SPECT) may also be promising, albeit not yet supported by prospective management outcome data. A prospective study comparing SPECT to CTPA and V/Q scan in non-pregnant patients is currently ongoing (NCT02983760), and SPECT may possibly be a promising technique in the future for pregnant women.

In spite of the optimization of CTPA protocols for pregnant women, the historical belief of significantly higher radiation doses to the mother's breast tissue of CTPA compared to V/Q scan still influences some physicians in their choice toward scintigraphy. Because of a very low likelihood of pulmonary comorbidity in this population, a two-step protocol is used in some centers: a perfusion scan is performed; PE is excluded in case of a normal perfusion scan. Ventilation sequences are only performed in case of an abnormal perfusion pattern to seek for a mismatch suggestive of PE. It should be noted that such a stepwise strategy has however not been validated in prospective trials. Caution is required in particular in the positive diagnosis of PE based on a perfusion scan alone, without having objectively confirmed that the perfusion abnormality is not associated with any parenchymal/ventilation abnormality.

The 2018 American Society of Hematology (ASH) guidelines for the diagnosis of VTE are highly driven by the willingness to avoid radiation even in the general population, and thus advocate for V/Q scan for patients likely to have a diagnostic scan and in centers where V/Q scans are available with expertise to interpret the results in a timely manner (14). The latest European Society of Cardiology (ESC) 2019 Guidelines provide specific recommendations for pregnant women. In terms of imaging test, their recommendation states “perfusion scintigraphy or CTPA with a low-radiation dose protocol” with a Class IIa, level C recommendation (13).

## Current Diagnostic Algorithms

As said before, to date, only two prospective management outcome studies have been published in pregnant women with suspected PE, reflecting the challenges of leading clinical trials in this setting.

### CT-PE Pregnancy Algorithm

The CT-PE pregnancy study published in 2018 (9) included 395 women with suspected PE and applied a diagnostic algorithm based on the sequential assessment of clinical PTP, D-dimer with the standard 500 ng/mL cutoff, lower limb venous CUS regardless of the presence of leg symptoms or signs, and CTPA as the first-line chest imaging technique (see Figure 1). PE prevalence was 7%, the failure rate of the strategy was 0.0% (95% CI 0.0–1.0%) and the percentage of patients managed without thoracic imaging 12%.

### ARTEMIS Algorithm

The ARTEMIS study published in 2019 (10) included 498 women with suspected PE and applied an adapted YEARS model (see Figure 1). PE prevalence was 4%, the failure rate of the strategy was 0.21% (95% CI 0.04–1.2%) and the percentage of patients managed without thoracic imaging 39%.

The detailed description of the respective strengths and limitations of these studies have been described elsewhere and are beyond the scope of this paper (15). The important message here is that such prospective outcome studies are gradually filling the knowledge gap in the optimal diagnostic management of pregnant women with suspected PE, and will certainly contribute to increase the appropriateness of these patient's management in the future.

## Remaining Controversies

Despite the recently published prospective data and evidence, controversies regarding the optimal diagnostic strategy for PE in pregnant women are still alive and the topic remains highly debated. As an example, the CT-PE pregnancy and ARTEMIS models have been challenged in an analysis performed on a UK cohort of 219 patients (DiPEP study) which includes pregnant women having PE diagnosed primarily by imaging. The authors concluded that both strategies were not safe (18). However, the original DiPEP study this retrospective analysis was performed on, suffered from many limitations. In particular, the DiPEP cohort was not a purely prospective cohort, different D-dimer tests with variable cutoffs were used, and there was no standardized diagnostic algorithm (27). The reported inferences from the recent analysis performed on this partly retrospective cohort are probably not as robust as the prospective management outcome trials, and the message advocating against D-dimer use based on this data should therefore be interpreted with caution.

Regarding the imaging test of choice, the major concern surrounding the use of any diagnostic test is the risk of maternal and fetal radiation exposure. While fetal exposure seems to be in the same range with both tests, CTPA is more radiating for the mother's breasts. Although no increased risk of early-onset breast cancer was observed in a large population cohort study with a median follow-up of almost 6 years after CTPA and of 7.3 years after V/Q scan, these findings might be considered as insufficiently reassuring, due to the limited length of follow-up (28). Also, the cumulative effect of repeated chest imaging is not well known. Importantly, the previously reported 12% rate of inconclusive CTPAs was not confirmed in the CT-PE pregnancy and in the ARTEMIS studies (reported rates of 7 and 0%, respectively) (9, 15), so that repeat chest imaging during the same diagnostic workup remains exceptional.

Despite these limitations, the risks associated with radiation exposure of both CTPA and V/Q scan are lower than the risk of missing a PE or of exposing unduly a pregnant woman to an anticoagulant treatment. As emergency access to V/Q scan is becoming difficult even in University Hospitals, CTPA will likely become the most used diagnostic test for most pregnant women with suspected PE who could not have the diagnosis excluded by PTP and D-dimer. Noteworthy, the radiation dose to the maternal breast with modern CTPA techniques is steadily decreasing and will probably reassure prescribing physicians in the near future. Ongoing prospective studies on this topic include the OPTICA study (NCT 04179487) whose aim is to assess the usefulness and safety of a low-dose CTPA protocol in pregnant patients with suspected PE (29).

In conclusion, despite the important recent advances in the field, there is room for further refinements and improvements of diagnostic strategies for suspected PE in pregnant women. Educational efforts should be strongly encouraged to pass the challenge of implementing validated diagnostic strategies in everyday clinical practice.

## Author Contributions

HR-E drafted the paper. GL and MR revised it critically for important intellectual content. All authors provided final approval of the version to be published.

## Conflict of Interest

The authors declare that the research was conducted in the absence of any commercial or financial relationships that could be construed as a potential conflict of interest. The handling editor, BT, declared a past collaboration with one of the author GL.

## Publisher's Note

All claims expressed in this article are solely those of the authors and do not necessarily represent those of their affiliated organizations, or those of the publisher, the editors and the reviewers. Any product that may be evaluated in this article, or claim that may be made by its manufacturer, is not guaranteed or endorsed by the publisher.
